# A Systematic Literature Review and Meta-Regression Analysis on Early-Life Energy Restriction and Cancer Risk in Humans

**DOI:** 10.1371/journal.pone.0158003

**Published:** 2016-09-19

**Authors:** Rachel J. J. Elands, Colinda C. J. M. Simons, Martien van Dongen, Leo J. Schouten, Bas A. J. Verhage, Piet A. van den Brandt, Matty P. Weijenberg

**Affiliations:** Department of Epidemiology, GROW School for Oncology and Developmental Biology, Maastricht University, Maastricht, the Netherlands; Flinders University, AUSTRALIA

## Abstract

**Background:**

In animal models, long-term moderate energy restriction (ER) is reported to decelerate carcinogenesis, whereas the effect of severe ER is inconsistent. The impact of early-life ER on cancer risk has never been reviewed systematically and quantitatively based on observational studies in humans.

**Objective:**

We conducted a systematic review of observational studies and a meta-(regression) analysis on cohort studies to clarify the association between early-life ER and organ site-specific cancer risk.

**Methods:**

PubMed and EMBASE (1982 –August 2015) were searched for observational studies. Summary relative risks (RRs) were estimated using a random effects model when available ≥3 studies.

**Results:**

Twenty-four studies were included. Eleven publications, emanating from seven prospective cohort studies and some reporting on multiple cancer endpoints, met the inclusion criteria for quantitative analysis. Women exposed to early-life ER (ranging from 220–1660 kcal/day) had a higher breast cancer risk than those not exposed (RR_RE all ages_ = 1.28, 95% CI: 1.05–1.56; RR_RE for 10–20 years of age_ = 1.21, 95% CI: 1.09–1.34). Men exposed to early-life ER (ranging from 220–800kcal/day) had a higher prostate cancer risk than those not exposed (RR_RE_ = 1.16, 95% CI: 1.03–1.30). Summary relative risks were not computed for colorectal cancer, because of heterogeneity, and for stomach-, pancreas-, ovarian-, and respiratory cancer because there were <3 available studies. Longer duration of exposure to ER, after adjustment for severity, was positively associated with overall cancer risk in women (*p* = 0.02). Ecological studies suggest that less severe ER is generally associated with a reduced risk of cancer.

**Conclusions:**

Early-life transient severe ER seems to be associated with increased cancer risk in the breast (particularly ER exposure at adolescent age) and prostate. The duration, rather than severity of exposure to ER, seems to positively influence relative risk estimates. This result should be interpreted with caution due to the limited number of studies and difficulty in disentangling duration, severity, and geographical setting of exposure.

## Introduction

Energy restriction (ER) without malnutrition has been reported to be the most effective dietary intervention to decelerate aging related diseases [1–4], including reductions in cancer risk in animal models of cancer. Lifelong ER starting early in life may be particularly effective in reducing cancer risk at a number of organ sites, predominantly on mammary tumours in rodents [5, 6].

Specific aspects of ER, such as the duration and the intensity of ER, may determine whether exposure is associated with an increased or decreased risk for different cancer sites in animal models [7, 8]. With regard to the duration of ER, the incidence of neoplasms was reduced following continuous ER throughout lifespan [5, 6, 9–24], whereas transient ER for several weeks followed by refeeding *ad libitum* has not consistently been associated with the same protective effect and may instead have adverse effects on carcinogenesis [15, 20, 22]. With regard to the intensity of ER, tumor incidence reduction starts becoming apparent at energy intake below approximately 80% of *ad libitum* levels in spontaneous- [18] and chemically induced tumor models [14]. Several studies have shown the tumor-inhibiting effect of ER to be dose-dependent[12] with the highest protection at about 60% of *ad libitum* energy intake[3, 4, 25] However, evidence exists for a transition phase of the ER effect: reversal from an increased to a decreased life- and health span [3, 4]. Energy intake reduction up to 65% improves life- and health span in rodents, most noticeably by reducing the incidence of multiple forms of cancer, yet it has been suggested that energy intake reduction higher than 65% could not impose the same health benefits [4].

As opposed to the results from controlled animal experimental studies, the scientific evidence for the relationship between ER and cancer risk in humans is inconclusive. Overweight is an established risk factor for many cancers and it is interesting to explore how ER, which is on the other end of the energy balance spectrum, is related to cancer risk, especially given the protective effects of life-long ER in animal models. Short-term experimental studies on voluntarily imposed ER in humans in combination with nutrient dense diets have been conducted to investigate physiological health effects in humans [26–28]. However, investigating long-term effects on cancer risk in human experimental studies is not ethical. Therefore, evidence for associations of ER with cancer in humans is only derived from observational studies. In these studies, ER exposure in humans is mostly early in life and often war-related. This complicates the matter since extreme conditions may be accompanied by other risk factors; such as stress [29], which may obscure the relationship. In addition, it is obvious that these extreme conditions do not translate directly into prevention, but evidence for such an association points to periods in life that are sensitive to energy balance and its effect on cancer risk decades later.

Existing reviews on human observational research concerning the association between early-life ER and cancer risk have been descriptive in nature. The association between ER in early-life and cancer risk in humans has neither been reviewed systematically nor has it been quantified. This is particularly true for site-specific cancers other than breast cancer. Evidence from *Elias et al*., *2005*, who found that overall cancer risk is pulled towards a positive association only when breast cancer cases were included in the analysis [22], further substantiates our objective to study site-specific associations. Therefore, we aimed to review the site-specific associations for ER and cancer risk or -mortality in the literature and, where possible, provide summary relative risk estimates. Comparison of the direction of the site-specific associations will provide insight into whether general or site-specific mechanisms might be involved in human cancer aetiology. Since most studies investigated ER in childhood and adolescence and later life cancer risk, we will focus on this time window. In addition, we aim to investigate in an explorative fashion, how contextual aspects of ER such as timing, duration and severity of early-life ER may impact the reported associations with cancer risk, as has been observed in animal studies.

## Methods

The literature was reviewed for human observational studies on ER in early-life, including adolescence and childhood, in relation to the site-specific cancer risk or mortality in later life until August 2015. PRISMA guidelines for publishing systematic reviews and meta-analysis were followed [30] (S1 Table). The review protocol is described below.

### Search strategy

PubMed and Embase were searched for full-text English-language papers on human observational studies combining the relevant keywords or medical subject headings as follows: ‘((energy restriction OR famine OR caloric restriction OR World War 2 OR World War II) AND (cancer risk) AND human)/ep)’. References cited in published original articles were hand-searched until no further studies were identified. Articles were selected only if an abstract was available.

### Study selection

Studies were included in the systematic literature review and meta-analysis if they met the following criteria: 1) study was conducted in a human population; and 2) outcome of interest was site-specific cancer risk or mortality, and effect estimates (hazard ratio (HR), risk ratio (RR) or odds ratio (OR)) with 95% confidence intervals (CIs) were reported or it concerned an ecological study. Studies exclusively on prenatal exposure to ER and ER due to anorexia nervosa were excluded.

### Data abstraction

#### Characteristics of included studies

Data were extracted from the included articles by one reviewer (RE). The following information was obtained from the included publications: the first author’s last name, publication year, study design, country of origin, cohort size, number of cases, number of person-years of follow-up, age or multivariable adjusted HRs, RRs or ORs and their corresponding 95% CIs, exposure contrasts, estimates of caloric intake, duration of ER, birth cohort, sex, age at exposure and cancer endpoints.

#### Methodological quality assessment of included studies

Qualitative assessment of the included cohort studies was examined according to the guidelines in the Newcastle-Ottawa scale (NOS) [31]. The NOS has been typically used for assessing the quality of non-randomized studies in meta-analyses. The NOS contains the following three subscales: selection of the study population (four items), comparability of exposed and non-exposed subcohorts (one item), and outcome assessment (three items). The following characteristics were evaluated: representativeness of the exposed cohort, selection of the non-exposed cohort, ascertainment of the exposure, demonstration that the outcome was not yet present at the start of the study, assessment of the outcome, follow-up time and completeness of follow-up. Quality of included studies was rated by two reviewers (RJJE, CCJMS). A third reviewer (MPW) was counselled in case of any disagreement. The NOS uses a star system to judge studies on key domains. For each domain either a ‘star’ or ‘no star’ is assigned, with a ‘star’ indicating the relevant study design aspect is considered adequate and unlikely to introduce bias. A cohort study can be awarded a maximum of eight stars.

### Meta-analyses

Pooled random effects and 95% CIs were estimated by the restricted maximum- likelihood estimator using the ‘metafor’ package for R statistical software environment (version 3.1.2) [32]. A random effects model was used, because the cancer (mortality) risk estimates found in the individual studies might be context dependent, due to study-specific characteristics such as duration and severity of ER. Therefore, variation in risk estimates between studies is expected to exceed chance (sampling error) variation, which is accounted for in a random-effects model. We pooled hazard ratios and risk ratios if at least three studies reported on cancer site-specific incidence or mortality and if Higgins’ index for between-study heterogeneity (I^2^) [33] in the reported effect sizes between studies was <50% [34, 35]. Heterogeneity was further tested using the Cochran's Q test (*p*< 0.1 indicates statistically significant heterogeneity). In case of statistically significant between-study heterogeneity, we decided to refrain from presenting the pooled relative risk estimate. For these cancer sites we restricted the results presentation to a forest plot visualizing the direction and strength of the associations. In the calculation of pooled effects, the contribution of each study was weighed by the inverse of its variance to take into account study specific variance and variance due to differences in sample size between the studies: $w_{i}=1/(v_{i}+{\hat{\tau }}^{2})$, where *v_i_* denotes the sampling variance (the square root of the standard error) for the given study and ${\hat{\tau }}^{2}$ denotes the estimate of (the total amount of heterogeneity between all studies) [32].

If studies were reporting on multiple categories of exposure to early-life ER, the outcomes for the most extreme exposure contrast were included in the meta-analysis. If a cohort reported effect estimates for multiple birth cohorts without an overall estimate, we first pooled estimates of these separate birth cohorts and included the pooled estimate in our meta-analysis. We did so, because the inclusion of multiple effect estimates from the same cohort for a particular endpoint will (artificially) lower the amount of heterogeneity between studies and will drive the pooled estimate into the direction of the findings within one particular cohort, especially in the event of few other cohort studies.

Following recommendations by Sterne et al., 2011, by default, publication bias was evaluated visually only if a minimum of 10 studies were available by inspecting the symmetry of funnel plots [36]. The degree of funnel plot asymmetry was assessed with the Egger's weighted regression test. Absence of publication bias is reflected in an intercept close to 0 with a corresponding *p*≥ 0.05 [37].

Subgroup analyses were conducted where possible for age of exposure to ER. Furthermore, in an explorative fashion, we studied three mixed-effects (meta-regression) models to elucidate whether ER severity and duration, which are inherently linked to the historical setting of the individual included cohort studies, explain part of the variability in effect estimates across studies. We included as explanatory (i.e. independent) variables ER severity, ER duration and ER severity and duration simultaneously, respectively. Since a meta-regression analysis is only advisable in the event of at least 10 individual studies (*i*.*e*. data points), these analyses were not performed for site-specific cancer outcomes, but all cancer outcomes in men and women respectively [38].

## Results

### Characteristics of included studies

**Fig 1:**
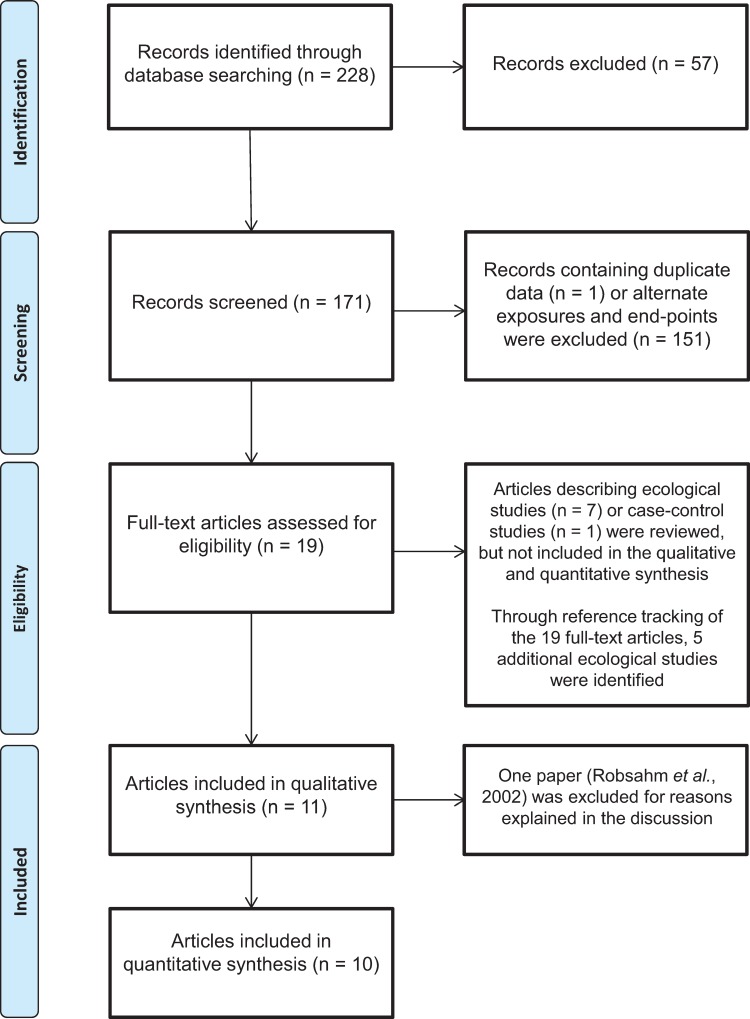
PRISMA flow diagram showing a breakdown of the study selection.

The flow chart of the search strategy is depicted in Fig 1. Electronic database search strategy retrieved 228 full-text articles which were all published in English. Fifty-seven review papers were excluded, leaving 171 records to be assessed for eligibility for the systematic review based on title and abstract. Subsequently, 151 records were excluded because the inclusion criteria were not met or because an exclusion criterion was fulfilled, e.g. papers exclusively on prenatal ER or anorexia nervosa as reported exposures. One study was excluded [39] because a more recent publication reported on the same association with longer follow-up time [40]. The nineteen remaining records referred to eleven publications on seven cohort studies, seven ecological studies and one case-control study, respectively. Reference-tracking of the nineteen included papers identified five additional ecological studies, resulting in twenty-four full-text articles that met the criteria for full review, some reporting on multiple cancer endpoints. Eight publications emanating from four cohort studies collected data from populations in Europe (two in the Netherlands, one in England and one in Norway) (Table 1) [40–46]. Three publications emanated from three cohort studies: one in China [47], one in Russia [7] and one from Israël [48]. One case-control study was based on a population from Israël [49] (Table 1). All twelve ecological studies investigated European populations (S2 Table) [50–62].

**Table 1:** Characteristics of included cohort and case-control studies.

Studies in chronological order of publication	Design	Country	Historical event	Intervention versus nonintervention cohort/arm (ascertainment of intervention)	Follow-up (years)	Completeness of follow-up	Cases (controls)	N cohort (subcohort)	Adjustments	End-points
Dirx et al., 1999 [42]	Case- cohort	The Netherlands	Dutch Hunger Winter	Lived in Western city vs. non-Western area (questionnaire)	6.3	>96%	1009	62,573 (1716)	^**a, b, c, d, e, f**^	Breast cancer risk
Dirx et al., 2001 [43]	Case- cohort	The Netherlands	Dutch Hunger Winter	Lived in Western city vs. non-Western area (questionnaire)	7.3	>96%	903	58,279 (1630)	^**a, d, e, f**^	Prostate cancer risk
Robsahm et al., 2002 [44]	Cohort	Norway	World War 2	Non-food versus food producing areas (registry)	28	-	7311	597,906	^**b, d, f**^	Breast cancer risk
Elias et al., 2004 [41]	Case-cohort	The Netherlands	Dutch Hunger Winter	Hunger vs. no hunger (questionnaire)	15.3 (median)	95%	585	15,396 (2352)	^**a, b, d, e**^	Breast cancer risk
Fentiman et al., 2007 [45]	Cohort	England	Occupation of Guernsey	Stayed vs. evacuated (questionarre)	15–20	-	97	2,377	^**a, b**^	Breast cancer risk
Koupil et al., 2009 [7]	Cohort	Russia	Siege of Leningrad	Lived in Leningrad vs. outside Leningrad (registry)	23–30	~95%	792	5330	^**a, b, c, d, e**^	Breast-, prostate-, stomach-, colorectal-, respiratory-, other cancers and all-site cancer mortality
Keinan-Boker et al., 2009 [48]	Cohort	Israël	Holocaust	Immigrated after the war vs. before the war (registry)	21	~93%	69,297	315,544	-	Breast-, prostate-, stomach-, colorectal-, lung and bronchial-, other cancers and all-site cancer risk
Hughes et al., 2010 [63]	Case- cohort	The Netherlands	Dutch Hunger Winter	Lived in Western city vs. non-Western area (questionnaire)	16.3	>96%	2971	120,852 (3981)	^**a, c, d, e, f**^	Colorectal cancer risk
Heinen et al., 2011 [46]	Case- cohort	The Netherlands	Dutch Hunger Winter	Lived in Western city vs. non-Western area (questionnaire)	13.3	>96%	446	120,852 (4774)	^**a, b, c, d, e, f**^	Pancreatic cancer risk
Schouten et al., 2011 [64]	Case- cohort	The Netherlands	Dutch Hunger Winter	Lived in Western city vs. non-Western area (questionnaire)	16.3	>96%	394	62,573 (2589)	^**a, b**^	Ovarian cancer risk
Li et al., 2012 [47]	Cohort	China	Chinese Famine during Great Leap Forward	Born between 1930–1964 vs. born between 1965–1999 (registry)	4	-	162	-	^**-**^	Stomach cancer mortality
Vin-Raviv et al., 2012 [49]	Case-control	Israël	Holocaust	Hunger vs. no hunger (structured interview)	-	-	65 (200)	-	^**d**^	Breast cancer risk

^a^ Anthropometric variables (body mass index and height)

^b^ reproductive variables (parity, age at first birth, age at menopause, hormone replacement therapy (never, ever), oral contraceptive use (ever, never), hysterectomy (yes, no))

^c^ smoking or alcohol consumption

^d^ socio-economic variables (education and economic status)

^e^ genetic factors (family history or genetic mutation tests) or

^f^ variables that indicate baseline energy consumption or energy expenditure (physical activity and energy intake)

Specific cancer (mortality) endpoints were reported for breast- [7, 41, 42, 44, 45, 48, 49, 53], prostate- [7, 43, 48, 56], colorectal- [7, 40, 48, 54, 57, 59], testicular- [50–52, 56, 58, 60], stomach- [7, 47], respiratory-/lung- [7, 48, 61], pancreas- [46], and ovarian [64] cancer, and multiple cancer sites [55] (Table 1 and S2 Table). Three studies calculated age standardized rate ratios and 95% confidence intervals by comparing the observed cancer rates in the exposed group with expected cancer rates in the general population, serving as an approximation for the risk in the non-exposed population [44, 47, 48]. Eight studies calculated hazard ratios [7, 40–43, 45, 46, 64], one relative risks[48], and one odd ratios [49].

### Exposure to energy restriction

All of the included prospective studies investigated exposures to war-related ER except for the Chinese study (Table 1) [47]. In most cohorts, exposure to ER was proxy-assessed using information on residential history during the war years from self-reports or registries [7, 40, 42–46, 48, 64]. In one study, exposure measurement was based on residential status and individual recall of severity of exposure to wartime ER [41]. A case-control study used interviewing techniques [49].

The estimated level of caloric intake was retrieved from historical references included in the prospective studies’ reports, and ranged from 220 kcal/day [48] to 1660 kcal/day [45] (S3 Table). With regard to ER severity, these historical references indicated either states of malnutrition [7, 40, 42, 43, 46–48, 64] or (semi-)malnutrition [45] during early-life ER. One cohort study from Norway reported moderate early-life ER with a nutritious balanced diet [44]. The duration of exposure to ER ranged from 5–6 months in the Netherlands Cohort Study on Diet and Cancer (NLCS) [40–43, 46, 64] to 72 months in the Jewish Cohort Study [48] (S3 Table).

### Methodological quality assessment of included cohort studies

Methodological quality assessment according to the NOS indicated that the total number of points assigned to each cohort study ranged between 6–7 on a 0–8 scale (S4 Table). Most studies failed to receive a point for the item ‘ascertainment of exposure”, which relates to the fact that most studies had to rely on proxy-assessment of war-related ER. Sensitivity analyses concerning the quality of the included studies were not conducted since the studies were comparable and of high quality.

### Association between early-life ER and site-specific cancer risk

**Fig 2:**
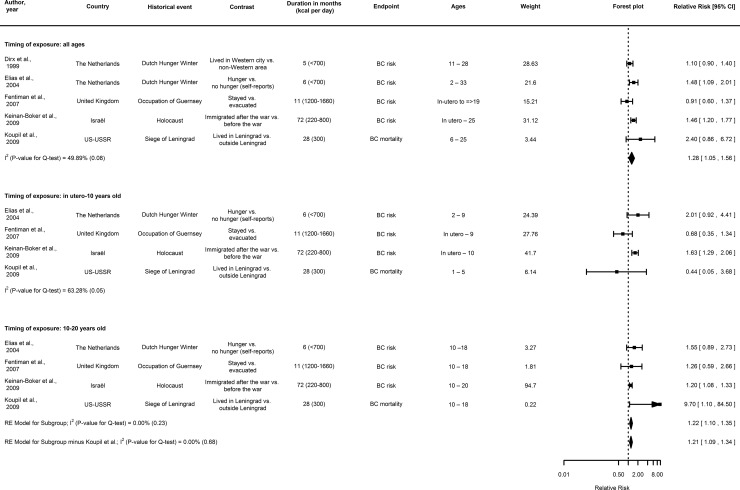
Forest plot showing a meta-analysis of cohorts on the association between transient early-life energy restriction and the relative risk and mortality of breast cancer, using the relative risk estimate as summary statistic. Note: Subgroup analyses were performed for childhood (in utero-10 years old) and adolescent (10–20 years old) exposure to ER in relation to breast cancer risk. If individual studies provided risk ratio estimates for different birth cohorts, these were pooled and the pooled estimate was taken along in the meta-analysis. Abbreviations: CI, confidence interval; BC, breast cancer.

**Fig 3:**
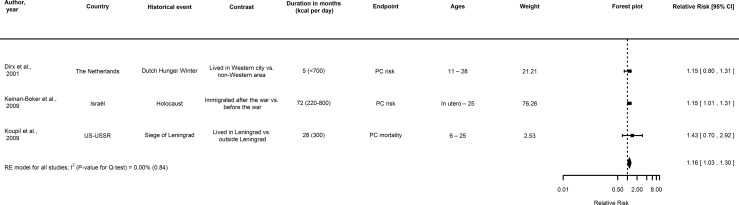
Forest plot showing a meta-analysis of cohorts on the association between transient energy restriction during (pre)adolescence and the relative risk and mortality of prostate cancer. Note: If individual studies provided risk ratio estimates for different birth cohorts, these were pooled and the pooled estimate was taken along in the meta-analysis. Abbreviations: CI, confidence interval; PC, prostate cancer.

**Fig 4:**
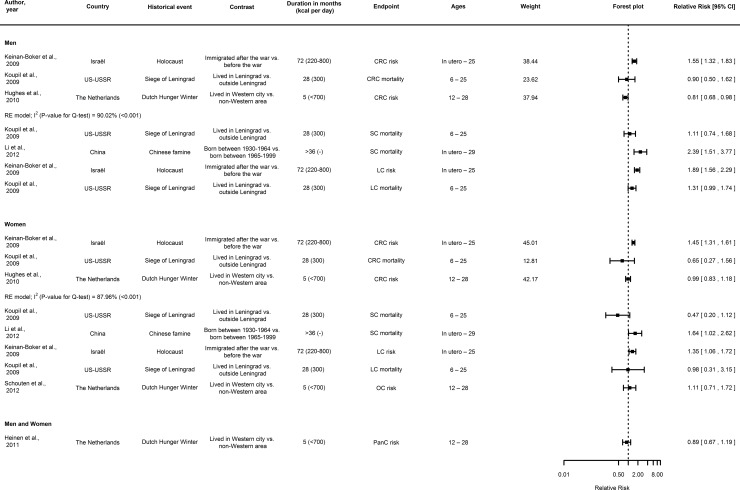
Forest plot showing a meta-analysis of cohorts on the association between transient energy restriction during (pre)adolescence and the relative risk and mortality at sites other than prostate cancer risk in males and breast cancer risk in females. Note: If individual studies provided risk ratio estimates for different birth cohorts, these were pooled and the pooled estimate was taken along in the meta-analysis. Abbreviations: CI, confidence interval; CRC, colorectal cancer; SC, stomach cancer; PaC, pancreatic cancer; LC, lung cancer; OC, ovarian cancer.

Three or more studies on ER and site-specific cancer risk were available for breast cancer (Fig 2), prostate cancer (Fig 3), and colorectal cancer in men and women (Fig 4), but not stomach, pancreatic and respiratory cancers in men and women, and ovarian cancer in women. For all sites, information on the risk ratios and hazard ratios extracted from the reports is provided in S5, S6 and S7 Tables.

#### Breast cancer

All but one of the five prospective cohort studies on early-life ER and breast cancer risk reported an association with increased risk of breast cancer although only significant in two studies (Fig 2 and S5 Table) [7, 41, 42, 45, 48]. Pooling the risk estimates for breast cancer from these five prospective cohort studies on ER between in utero—33 years of age showed a significantly increased risk (RR_RE_ = 1.28, 95% CI: 1.05–1.56, I^2^ = 49.89%; *p* = 0.08 for Cochran’s Q test) (Fig 2). ER exposure was between 220–1660 kcal/day. A meta-analysis could also be conducted for ER exposure between 10 and 20 years of age as shown in Fig 2. Women exposed to ER between 10 and 20 years of age had significantly increased risk of breast cancer compared to those not exposed during that age period (RR_RE_ = 1.21, 95% CI: 1.09–1.34, I^2^ = 0% for Cochran’s Q, *p* = 0.68). We refrained from pooling relative risk estimates for ER exposure between 0–10 years of age, because the Cochran's Q test indicated statistically significant between-study heterogeneity (I^2^ = 63.28%; *p* = 0.05 for Cochran’s Q) (Fig 2). The study by *Robsahm et al*.,[44] could not be included in the meta-analysis since for the exposure contrast that was investigated, i.e. non-food versus food producing areas, there were already differences in absolute cancer incidence that already existed before war-related exposure occurred. The findings supported an increased breast cancer risk among birth cohorts that were of adolescent age and living in non-food producing areas during WWII, who were exposed to ER compared to food-producing areas. One case-control study on early-life ER during the Holocaust and breast cancer risk reported an increased risk for ER exposed women [49]. In contrast, two ecological studies reported findings suggesting an inverse association for ER with breast cancer incidence [53] and mortality (S2 Table) [55]. A drop in breast cancer incidence rates was observed in Norwegian women exposed to war-time related ER (intake approx. 20% restricted [65]) during puberty [53]. Similarly, breast cancer mortality was low in women in early post-war Germany but increased afterwards comparable to levels in the United States. These women born around the war years in Germany were restricted to an estimated 1412–1600 kcal/day intake in 1945 when food supplies were plummeting (S2 Table) [55].

#### Prostate cancer

Three prospective cohort studies on early-life ER and prostate cancer risk and mortality indicated that men exposed to ER have a higher prostate cancer risk compared to those not exposed [7, 43, 48], although only significant for one study. Results from the meta-analysis indicate that men exposed to ER (energy intake estimates ranging from 220–800 kcal/day) had a significantly increased prostate cancer risk compared to non-exposed men (RR_RE_ = 1.16, 95% CI: 1.03–1.30; I^2^ = 0%; *p* = 0.84 for Cochran’s Q) (Fig 3 and S6 Table).

In contrast, two ecological studies reported findings suggesting an inverse association between ER and prostate cancer incidence [56] and mortality [55] (S2 Table). One ecological study was conducted in Denmark and reported a low point in prostate cancer incidence after the Second World War [56] in individuals potentially subjected to an estimated 7% reduction in energy intake [65] (S2 Table). Similarly, prostate cancer mortality was low in males in early post-war Germany, but increased afterwards comparable to levels in the United States; men were subjected to an estimated 1412–1600 kcal/day in 1945 compared to those borne earlier or later [55] (S2 Table).

#### Colorectal cancer

Three cohort studies on colorectal cancer reported positive (in men and women) [48], inverse (in men only) [40] and null associations [7] with early-life ER (Fig 4 and S7 Table). We refrained from pooling the risk estimates for colorectal cancer from these three prospective cohort studies on ER, due to statistically significant between-study heterogeneity in men (I^2^ = 90.02%; *p*< 0.001 for Cochran’s Q test), (Fig 4) and women (I^2^ = 87.96%; *p*< 0.001 for Cochran’s Q test), (Fig 4). The study on childhood and adolescent ER during the Holocaust reported associations with increased colorectal cancer risk in both men and women [48]. One prospective cohort study was on adolescent ER during the Dutch Hunger Winter and its association with proximal, rectal and overall colorectal cancer incidence demonstrating an association with decreased colorectal cancer risk in men, but no association in women (Fig 4 and S6 and S7 Tables) [40]. In the study on childhood and adolescent ER during the siege of Leningrad, a non-significant decreased colorectal cancer risk was observed in both men and women [7].

Two ecological studies indicated a drop in age-standardized incidence for colorectal cancer in birth cohorts encompassing the period of the Second World War in Norway, Sweden, Denmark and Estonia, but this drop in estimated colorectal cancer incidence did not extend to Finland (S2 Table) [57, 59]. The drop in absolute colorectal cancer incidence in Norway was most pronounced for localizations in the proximal colon for the birth cohorts 1939–1948 for men and 1944–1953 for women [57]. Also, men and women born in Norway between 1944 and 1948 seemed to have a lower risk for cancer of the distal colon and rectum than was expected on the basis of the general trend [57]. An ecological study conducted in Sweden, reported that the relative risk of right-sided colon cancer leveled off in men and women born after 1930, whereas left-sided colon cancer incidence was constant in cohorts born until 1930 and decreased later (S2 Table) [54].

#### Stomach cancer

There are two prospective studies on early-life ER and stomach cancer mortality (Fig 4 and S7 Table). The first study on childhood and adolescent ER and stomach cancer mortality during the siege of Leningrad reported null associations for both men and women [7]. The second study on childhood ER during the Chinese economic depression and stomach cancer mortality observed a positive association in both men and women [47].

#### Pancreatic cancer

One cohort study on adolescent exposure to ER during the Dutch Hunger Winter and pancreatic cancer risk reported no associations in men and women (Fig 4) [46].

#### Lung cancer

There are two cohort studies reporting on early-life ER and lung cancer risk (Fig 4 and S7 Table). The study on childhood and adolescent ER during the Holocaust and lung cancer risk showed associations with increased lung cancer risk in both men and women[48] The study on childhood and adolescent ER and lung cancer mortality during the siege of Leningrad showed null associations in both men and women [7]. One ecological study showed an increased lung cancer risk in men and women born during or after the Second World War in Austria; overall, there was a decreasing risk in men, but not women, with increasing birth year [61]. However, it is difficult to disentangle changes in smoking habits from other exposures, e.g. starvation [61].

#### Testicular cancer

Age-period-cohort analyses in ecological studies have indicated reduced testicular cancer incidence rates, interrupting a trend of increasing incidences over time, for cohorts born during the Second World War in Norway, Sweden, and Denmark, but not in Finland (S2 Table) [50–52, 56, 58, 60].

#### Ovarian cancer

One cohort study on adolescent exposure to ER during the Dutch Hunger Winter and ovarian cancer risk showed no association in women (Fig 4 and S7 Table) [64].

### Duration, severity and timing of ER

**Fig 5:**
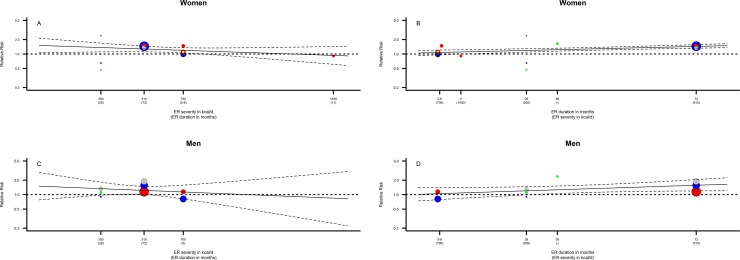
An overview of some of the contextual aspects of energy restriction that might modulate the association of early-life energy restriction with cancer risk. Note: The estimated caloric intake (in units of 100 kcal/day) was based on the mid-point caloric intake reported in the publications and was plotted against the reported relative risk ratios from the individual studies separately for women (panel A) and men (panel C). The estimated duration of ER (in months) was plotted against the reported relative risk ratios from the individual studies separately for women (panel B) and men (panel D). In women, the data points indicated in red represent studies reporting on breast cancer risk or mortality; the data points indicated in blue represent studies reporting on colorectal cancer risk or mortality; the data points indicated in green represent studies reporting on stomach cancer risk or mortality; the data points indicated in grey represent studies reporting on lung cancer risk or mortality; and the data points indicated in yellow represent a report on ovarian cancer risk. In men, the data points indicated in red represent studies reporting on prostate cancer risk or mortality; the data points indicated in blue represent studies reporting on colorectal cancer risk or mortality; the data points indicated in green represent studies reporting on stomach cancer risk or mortality; and the data points indicated in grey represent studies reporting on lung cancer risk or mortality. The dashed lines indicate the confidence intervals of the meta-regression line.

The contextual aspects of ER such as duration and severity of early-life ER are an inherent characteristic of the individual studies and these contextual aspects may impact the reported associations between early-life ER and cancer risk. Due to the limited number of studies available it was not possible to disentangle these effects for the different cancer sites separately. To estimate whether between-study heterogeneity was explained by the covariates duration of ER and severity of ER a mixed-effects meta-regression model was fitted across all cancer sites for men and women. A longer duration of exposure to early-life ER (in months) was associated with a (borderline) increased overall cancer risk in men (*p* = 0.07) and women (*p*< 0.001) (Table 2 and Fig 5). The associations were statistically significant after adjusting for severity of exposure in women (*p*< 0.001) but not in men (*p* = 0.08) (Table 2). Particularly, in women, adding duration of ER to the model substantially reduced heterogeneity between cohort studies in the meta-analysis (Table 2). Severity of ER was not associated with the reported effect size in cohort studies in men (*p* = 0.54) and women (*p* = 0.20) (Table 2 and Fig 5). Yet, overall cancer risk in women tended to increase as the caloric intake per day decreased (Table 2 and Fig 5).

**Table 2:** Meta-regression for exposure to early-life energy restriction and all type cancer risk/mortality including moderators.

Endpoint	Mixed-effects model unless otherwise specified	Beta intercept	95% CI	Beta	95% CI	I^2^	R^2^	Test for heterogeneity	Test for residual heterogeneity	Test of moderators
								*p*-value	*p*-value	*p*-value
All cancers women	RE model			0.20	(0.07, 0.34)	57.41%		0.002		
	RR ~ severity of exposure	0.41	(0.06, 0.76)	-0.03	(-0.08, 0.02)	48.06%	37.40%		0.01	0.20
	RR ~ duration of exposure	0.05	(-0.07, 0.17)	<0.01	(0, 0.01)	0.02%	99.99%		0.15	<0.001
	RR ~ severity of exposure +	0.10	(-0.31, 0.51)	-0.01	(-0.06, 0.04)	0.00%	100.00%		0.11	<0.001
	duration of exposure			<0.01	(0, <0.01)					
All cancers men	RE model			0.26	(0.06, 0.46)	84.42%		<0.001		
	RR ~ severity of exposure	-		-		83.01%	0.00%		<0.001	0.54
	RR ~ duration of exposure	-0.01	(-0.30, 0.33)	0.01	(>-0.01, 0.01)	77.73%	29.26%		<0.001	0.07
	RR ~ severity of exposure +	0.05	(-0.63, 0.73)	<0.01	(>-0.01, <0.01)	73.92%	36.64%		<0.001	0.08
	duration of exposure			0.01	(0, 0.01)					

Note: The unit increases in severity of exposure and duration of exposure were 100 kilocalories per day and months, respectively; severity and duration of exposure were inversely correlated in women and men (r = -0.36 and -0.12, respectively; *p* = 0.24 and 0.76, respectively). Abbreviations: CI, confidence interval; RE model, random-effects model; RR, relative risk.

* Estimates are not shown, because the test of moderators was not statistically significant

## Discussion

The epidemiological evidence for a sustained effect of transient (pre)adolescent ER on site-specific cancer risk has been inconclusive and not been reviewed or quantified previously. In this systematic review and meta-analysis of observational studies, the pooled results of cohort studies indicate that women exposed to ER (energy intake ranging from 220–1660 kcal/day) during childhood and adolescence have a 28% increased breast cancer risk. Also, pooled results from cohort studies indicate that exposure to ER (energy intake ranging from 220–800 kcal/day) during childhood and adolescence is associated with a 16% increased risk of prostate cancer. Summary risk estimates for colorectal-, stomach-, pancreatic-, ovarian- and respiratory cancer could not be calculated due to the limited number of studies available or study heterogeneity. Meta-regression analyses were conducted across all cancer sites and suggested that a longer duration of exposure (in months) to early-life ER is (borderline) associated with increased cancer risk in women and men. Particularly, in women, between-study heterogeneity was explained by the duration of early-life ER. The associations remained statistically significant in women after adjusting for severity of exposure.

Of note is that the results from the meta-regression analysis are exploratory and should be interpreted with caution given that for women only 6 cohorts with 13 risk estimates were included, and for men only 4 cohorts with 10 risk estimates, resulting in a limited power to discriminate between different covariates. The meta-regression analysis showed that the effect sizes in women tended to increase with a decrease of daily caloric intake. This trend was not significant, however. The lack of cohort studies that have investigated more moderate exposures to early-life ER may have obscured a possible relation.

### Inconsistencies between human observational studies

The most obvious finding emerging from this review is the inconsistency of the observed associations between early-life ER and site-specific cancer incidence obtained from various types of human observational studies. Ecological studies suggest either no effect or decreased site-specific cancer risk after transient exposure to severe early-life ER, whereas, prospective cohort studies suggest no effects or increased site-specific cancer risk. There are several potential reasons for the discrepancies between observational studies such as the unique historical contexts and residual confounding from baseline geographical differences in cancer incidence and from other exposures related to war-related uncontrolled ER.

The unique historical settings of the observational studies are associated with geographic location and with the duration and severity of ER. Certain aspects of ER, i.e. the timing of exposure [42, 43, 66, 67], its duration and/or severity [22, 48, 49], may determine whether ER is associated with an increased or decreased risk for different cancer sites. Animal studies have indicated that continuous ER may be particularly effective in reducing cancer risk when started early in life [3]. Our meta-analysis indicated that women exposed to severe transient ER between 10–20 years of age were at increased risk of breast cancer, whereas no consistent associations were observed for women exposed between 0–10 years of age. Particularly adolescence has been suggested to coincide with a period in which the developing mammary gland is sensitive to environmental signals [68, 69]; this has also been observed for exposure to nutritional stimuli, for example, transient severe ER [42, 66, 70, 71]. Regarding the duration of early-life ER, evidence from animal studies indicated that transient ER followed by refeeding *ad libitum* may have adverse effects on carcinogenesis[12, 20, 22] as opposed to continuous ER [18, 24]. Most human studies investigated transient exposures to early-life ER; and in some studies, but not all, reduced food intake persisted for several years after ER exposure [7, 47–49]. Also there is evidence concerning the severity of ER; a transition phase of ER may exist between 40% to 65% of daily regular caloric intake, at which the effect of ER reverses from an increase to a decrease of life and health span [3, 4]. Typically, the exposures to early-life ER in prospective studies were severe (energy intake estimates ranging from 220–1660 kcal/day, corresponding with a reduction in daily energy intake compared to current common daily allowances of 2,000 kcal in adults ranging from 17–89%) and coincided with severe ER (>40%) in all but one of the studies [45]. In contrast, ecological studies investigated exposures to moderate ER that were mainly experienced in Denmark, Norway, Sweden and Finland where populations were exposed to an estimated reduction of 4–20% or less of daily caloric intake [65], accompanied by a nutritionally balanced diet [50–54, 56–60]. Even though in ecological studies individual data on exposure of the cancer cases are lacking, it can be assumed that the observed reductions in anthropometric measures, *e*.*g*. weight and height, during the WW-II years in Europe are approximately reflecting the prevailing nutritional conditions in those countries [65, 72]. The inverse associations between ER and cancer risk found in ecological studies suggest that moderate ER with adequate nutritional balance could exert a protective effect on cancer whereas more extreme exposure to ER, as reported in prospective cohort studies, might convey a higher cancer risk. This suggestion is supported by a study examining the effects of long-term moderate caloric intake reduction in children and adolescents in Pre-War Britain that resembles the evidence from human ecological studies and animal experimental models that continuous moderate ER may exert a protective effect on cancer mortality [73].

Another potential reason for the difference in findings between observational studies is that many prospective studies do no account for existing baseline differences in absolute cancer incidence across exposure groups. In prospective studies, often a geographical contrast within a country, *e*.*g*. food-producing ‘rural’ areas versus non-food producing ‘urban’ areas, was employed as a proxy for unrestricted vs. restricted energy intake [7, 39–44, 46, 64]. These geographical contrasts may include longstanding differences in absolute cancer incidence that existed already before the war-related exposure occurred. For example, *Robsahm et al*. [44] observed a higher cancer incidence in urban areas as compared to rural areas. These geographical differences in cancer incidence may partly result from the different distribution of cancer related risk factors. Since, ecological studies applied temporal contrasts inferred from age-period-cohort modelling these studies were not impacted by geographical differences in absolute cancer incidence; this might explain in part the contrasting findings from ecological and prospective cohort studies. Longstanding baseline differences in cancer risk between geographical areas (*i*.*e*. urban and rural areas) often coincide with the groups that are contrasted in terms of ER. This may mask a true effect of ER on outcome and may thus have caused attenuation of any true inverse associations that may now remain unobserved or even be reversed revealing positive associations. This potential bias may have resulted in the observation that ER is accompanied with an increased risk of breast and prostate cancer in the meta-analysis. Therefore, caution is warranted in interpreting the results from observational epidemiologic studies on early-life ER in relation to cancer.

Furthermore, exposure to war-related ER is potentially accompanied with other risk factors for cancer, such as stress, which may explain the observed positive associations between more severe early-life ER and cancer risk, and thereby contribute to the difference in findings between cohort and ecological studies. For example, it has been reported that post-traumatic stress disorder in exposed Jewish children during Holocaust suffering from severe ER was associated with increased breast cancer risk [29].

### Mechanistic evidence

Some findings from the limited number of animal studies that have investigated the cancer-related effects of transient severe ER early in life followed by *ad libitum* food consumption are supportive[12, 20, 22] of the null and positive findings from human prospective studies. Still, while animal experimental studies find inverse associations, in some cases, such as the Dutch Hunger winter, the counteracting increased caloric intake following the famine, might have obscured associations. Whereas the food availability after the war recovered quickly in the Netherlands [74, 75] and Norway [76, 77], constraints in food availability sustained during the post-war period in the Soviet Union [78]. It has been argued that transient severe ER followed by acute access to abundant food imposes an overshoot of mitogenic growth hormone factor signaling [79], through the growth hormone-insulin-like growth factor (GH-IGF) axis and may result in a modest acceleration of the carcinogenic response in animals [21] and humans [79]. In contrast, continuous moderate ER enables the body’s metabolism to adapt on the long-term by responding with lower circulating IGF-1 [3, 80, 81] and upregulation of IGF binding protein (IGFBP)-1 levels [82] which may suppress carcinogenesis. In general, together with the hypothalamic-pituitary-gonadal axis [67, 83], the GH-IGF axis coordinates growth and development early in life, a time during which serum levels of these hormones peak under *ad libitum* conditions [84]. When ER occurs early in life, a period in which development and appropriate functioning of the reproductive axis demands a fixed quantity of energy stores [83], these axes might be permanently modified, influencing cancer risk later in life. Yet, for the GH-IGF-1 axis it is known that the response to ER is different between species. Whereas in both rodents and humans, serum IGF-1 levels decrease [85] and result in a concomitant reduction in growth hormone (GH) serum levels in rodents, GH serum levels tend to increase in humans [82, 86]. The contrasting fasting response between species may lead to differences in the observed associations between early-life ER and cancer risk in humans and animal models of carcinogenesis. Correspondingly, an experimental study in humans with a two-year caloric intake restriction of 30% from ad libitum, which resembled the controlled setting of moderate ER with nutrient dense diets in animal experiments, observed physiological changes similar to those in caloric restricted rodents, with the exception for IGF-1 and GH serum levels [26–28]. This suggests that the mechanisms linking early-life ER to cancer risk in animal experimental models of cancer cannot directly be extrapolated to humans.

### Future directions for human observational research

It seems that a negative energy balance in childhood and adolescence may impact on cancer occurring much later in life. However, the heterogeneity of observational studies to date makes it difficult to draw conclusions. This raises the question on how to proceed in this field. Molecular epidemiological approaches within existing studies may contribute to better insight into the mechanisms that may be at play. However, epidemiologic data regarding the mechanisms underlying an association between early-life ER and human site-specific cancer risk are scarce, because exposure to ER is rarely available in observational studies and few studies are large enough to allow for small subgroup analyses. In addition, tissues and molecular markers to investigate mechanisms are not commonly available. Tumor material, stored in pathology labs, can offer new opportunities for ongoing large-scale epidemiological studies since the tumors may provide molecular signatures of a carcinogenic process that started years ago.

Epigenetic changes are thought to be an early step in the carcinogenic process, typically environmental influences on epigenetics are most prominent during childhood and adolescence, the time frame of susceptibility to epigenetic/transcriptional modulations that undergo establishment and maturation [87, 88]. These epigenetic patterns can persist throughout life when occurring in stem cells [89]. Epigenetic markers can therefore be employed as a molecular signature to study how environmental exposures early in life may induce persistent epigenetic changes that influence methylation patterns in cancer occurring much later in life [90]. Hypermethylation through the CpG island methylator phenotype (CIMP) in the promotor region of specific cancer-related genes is considered an early event in carcinogenesis[91, 92] and associations between early-life indicators of energy balance and CIMP in CRC may exist in particular. ER in adolescence has been inversely associated with CRC CIMP phenotype [93] which suggests that exposure to a transient environmental condition during this period of life can lead to sustained epigenetic modifications that impact cancer risk in adult life. Early-life ER has also been inversely associated with the risk of having a colorectal tumor characterized by IGFBP methylation [94]. Even though these types of molecular epidemiologic data are scarce, they are supportive of an inverse association between early-life ER and the risk of colorectal cancer. Therefore, replication of these studies and extension to other sites and mechanisms are needed to further substantiate the evidence.

## Conclusion

In general, it seems that severe transient ER in the absence of a nutritious diet is associated with increased cancer risk in the breast (for ER exposure at adolescent age) and prostate. Evidence for associations between severe transient ER early in life and risk at other cancer sites is limited. In the meta-analysis of the prospective cohort studies, the duration, rather than severity of exposure to early-life ER, seems to positively influence relative risk estimates. Results should be interpreted with caution due to the limited number of studies and difficulty in disentangling duration, severity and geographical setting of the exposure. For exposure to less severe ER, a decreased association with cancer risk is generally observed, although this is derived only from ecological studies. This raises the question on how to proceed in this field. Molecular epidemiological approaches within existing studies may contribute to explain in part the variation in disease risk across sites providing better insight into the mechanisms that might be at play.

## Supporting Information

S1 Table PRISMA checklist. (PDF)

S2 Table Overview of characteristics of ecological studies describing birth cohort trends in cancer incidence and cancer mortality in the period encompassing the Second World War. (DOCX)

S3 Table Study characteristics concerning the duration and caloric intake as reported by the included cohort studies. (DOCX)

S4 Table Qualitative assessment of included cohort studies according to the quality subscales of the Newcatle-Ottowa scale. (DOCX)

S5 Table Overview of cohort studies investigating transient energy restriction in early-life and breast cancer mortality and incidence in later life. (XLSX)

S6 Table Overview of cohort studies investigating transient energy restriction in early-life and prostate cancer mortality or risk in males. (XLSX)

S7 Table Overview of cohort studies investigating transient energy restriction in in early-life and cancer mortality or risk at sites other than prostate cancer risk in males and breast cancer risk in females. (XLSX)
