# Supplementary material for: A Systematic Literature Review and Meta-Regression Analysis on Early-Life Energy Restriction and Cancer Risk in Humans
Source: PLoS One. 2016 Sep 19;11(9):e0158003. doi: 10.1371/journal.pone.0158003 (PMC5028056; doi:10.1371/journal.pone.0158003)
Supplement: S2 Table — (DOCX) [file pone.0158003.s002.docx]

**S2 Table:** Overview of characteristics of ecological studies describing birth cohort trends in cancer incidence and cancer mortality possibly connected to energy restriction in the period encompassing World War II.

| **Study^a^** | **Country** | **Estimated ER**^a^ | **N cases** | **(Poisson) model** | **Birth Cohorts** |
| --- | --- | --- | --- | --- | --- |
| **Breast cancer incidence** |  |  |  |  |  |
| *Tretli et al., 1996* | Norway | 20%^b^ | 20,111 | Age-period-cohort model and exposure/sensitivity model | 1903-1953 |
| **Prostate cancer incidence** |  |  |  |  |  |
| *Moller et al., 2001* | Denmark | 7%^b^ | NA | Age-period-cohort model | 1858-1948 |
| **Colorectal cancer incidence** |  |  |  |  |  |
| *Thörn et al., 1998* | Sweden | 4%^b^ | Colon cancer: 85,547; rectal cancer: 49,096 | Age-period-cohort model by gender | 1875-1974 |
| *Svensson et al., 2002* | Norway | 20%^b^ | Men: 32,981; women: 32,812 | Age-period-cohort model by gender | 1874-1953 |
| *Svensson et al., 2005* | Norway (NO), Sweden (SE), Denmark (DK), Finland (FI), Iceland (IC), Estonia (ES) | NO: 20%; FI: 17%; DK: 7%; SE: 4%; IC: NA; ES; NA ^b^ | NO: 61,836 ; FI: 35,003; DK: 88,832 ; SE:119,416; IC: 1,762; ES: 10,224 | Age-period-cohort model by gender | 1874-1953 |
| **Testicular cancer incidence** |  |  |  |  |  |
| *Wanderås et al., 1995* | Norway | 20%^b^ | 3,927 | Age-period-cohort model | 1916-1970 |
| *Bergström et al., 1996* | Denmark (DK), Norway (NO), Sweden (SE), East Germany (E-GER), Finland (FI), Poland (PL) | DK: 7%; NO: 20%; SE: 4%; E-GER: NA; FI: 17%; PL: NA^b^ | DK: 6,352; NO: 3,111; SE: 3,770; E-GER: 10,051; FI: 1,174; PL: 6,450 | Age-period-cohort model | 1860-1969 (DK, fewer birth cohorts in other countries) |
| *Moller et al. 1989; Moller et al., 2001* | Denmark | 7%^b^ | NA | Age-period-cohort model | 1888-1978 |
| *Richiardi et al., 2004* | Denmark (DK), Estonia (ES), Finland (FI), Latvia (LAT), Lithuania (LIT), Norway (NO), Poland (PL), Sweden (SE) | DK: 7%; ES: NA; FI: 17%; LAT: NA; LIT: NA; NO: 20%; PL: NA; SE: 4%^b^ | DK: 9,138; ES: 293; FI: 1,842; LAT: 392; LIT: 259; NO: 4,888; PL: 4,388; SE: 5,830 | Age-period-cohort model for DK, FI, NO and SE, which had a sufficiently long period of registration and a large number of cases | 1885-1980 (DK, fewer birth cohorts in other countries) |
| *Jacobsen et al., 2006* | Denmark (DK), Finland (FI), Norway (NO), Sweden (SE) | DK: 7%; FI: 17%; NO: 20%; SE: 4%^b^ | NA | Age-standardized rates and incidence rates over age by birth cohort | 1938-1983 |
| **Lung cancer mortality** |  |  |  |  |  |
| *Borsoi et al., 2011* | Austria | NA | NA | Age-period-cohort model by gender | 1880-1960+ |
| **Site-specific cancer mortality** |  |  |  |  |  |
| *Becker al., 2001* | West-Germany versus United States of America (USA) | ~40-50%^c^ | NA | Age-standardized mortality rates by gender | NA |

Abbreviations: CRC, colorectal cancer; NA, not available.

^a^ The main results from these studies are described in the manuscript and full references are included at the end of the manuscript.

^b^ Angell-Andersen et al. (2004). Ann Hum Biology*,* 31(3): 342-355. DOI:10.1080/03014460410001685304. In addition, Lund Nilsen and Vatten (2001) report intakes of 2700 kcal/day during World War 2 versus 3475 kcal/day pre-war in Norwegian families (Br J Cancer, 85(7): 959-961. DOI: 10.1054/ bjoc.2001.1946).

^c^ Becker et al., 2001 give the following description: while in 1937 the average calorie demand per capita was assessed to be 2,750 kcal per day, the food allocated by the food ration cards led to a decreasing calorie supply, reaching a nadir in 1945 with estimated 1,412-1,600 kcal per capita per day.
