# Supplementary material for: A Systematic Literature Review and Meta-Regression Analysis on Early-Life Energy Restriction and Cancer Risk in Humans
Source: PLoS One. 2016 Sep 19;11(9):e0158003. doi: 10.1371/journal.pone.0158003 (PMC5028056; doi:10.1371/journal.pone.0158003)
Supplement: S3 Table — (DOCX) [file pone.0158003.s003.docx]

**S3 Table:** Study characteristics concerning the duration and caloric intake as reported by the included cohort studies.

| Cohort^a^ | Country | Historical event | Intervention period in months (daily caloric intake) | Additional notes^a^ |
| --- | --- | --- | --- | --- |
| *Elias et al, 2004* | The Netherlands | Dutch Hunger Winter | 6 (<700 from January 1945 onwards) | The diet remained nutritionally balanced, but the population living in western urban areas of the Netherlands experienced a rationing of <700 kilocalories per capita per day from January 1945 onwards (*Burger et al., 1948; Dols et al., 1946*). |
| *Fentiman et al, 2007* | England | Occupation of Guernsey | 11 (1200-1660) | From June 1944 onwards, the island’s food supplies were severely limited to approximately 1200 kilocalories per capita per day, which lasted until May 1945, although some Red Cross parcels brought relieve (~460 kilocalories) from December 1944 onwards (*Fentiman et al., 2007*). |
| *Keinan-boker et al, 2009* | Israël | Holocaust | 72 (220-800) | Jews experienced a long-term and severe energy restriction due to being interred in concentration camps and ghettos at the beginning of World War 2, often resulting in malnutrition and associated clinical manifestations, *e.g.* rickets, night blindness, anemia, and scurvy (*Keinan-Boker et al., 2009*). |
| *Koupil et al, 2009* | Russia | Siege of Leningrad | 28 (300 in winter 1941-42) | The siege of Leningrad lasted from September 1941 to January 1944 (28 months), leading to an acute food shortage and severe malnutrition, with its peak in the winter of 1941-42 when ~300 kilocalories per capita per day were provided (*Koupil et al., 2009*). |
| *Robsahm et al, 2009* | Norway | World War II | 60 (20% restricted) | The diet remained nutritionally balanced during the war years in Norway, but was 20% restricted (*Angell-Andersen et al., 2004; Robsahm et al., 2009*). |
| *Li et al, 2012* | China (Zhaoyuan County) | Chinese famine during ‘’Great Leap Forward’’ | >36 with a 24-month peak (-) | The famine affected all of China, but its severity and duration, including the beginning and end, varied across regions, and, therefore, the famine is difficult to define for any given area with precision; the Chinese famine followed the already widespread presence of chronic undernutrition (*Huang et al. 2010; J Nutr, 140:1874–1878. DOI:10.3945/jn.110.121293*). The severity of exposure for a particular county was estimated by the cohort size shrinkage estimate, which took into account the mean cohort size of a person born during the famine and of a person born immediately before and after the famine. This shrinkage estimate indicated that Zhaoyuan County had a value near the highest of famine indexes for 35 counties; in addition, the duration of the famine in Zhaoyuan County was estimated to be longer than average (*Huang et al. 2010; J Nutr, 140:1874–1878. DOI:10.3945/jn.110.121293; Li et al., 2012*). |
| *Dirx et al, 1999, Dirx et al, 2001, Hughes et al, 2010, Heinen et al, 2011, Schouten et al, 2011* | The Netherlands | Dutch Hunger Winter | 5 (<700 from January 1945 onwards) | See *Elias et al., 2004*. The height of the Dutch famine has been described to have been from December 1944 until April 1945, lasting approximately 5 months (depending on the exact definition). |

^a^ References not given in full are available in full in the manuscript.
