# Supplementary material for: A Systematic Literature Review and Meta-Regression Analysis on Early-Life Energy Restriction and Cancer Risk in Humans
Source: PLoS One. 2016 Sep 19;11(9):e0158003. doi: 10.1371/journal.pone.0158003 (PMC5028056; doi:10.1371/journal.pone.0158003)
Supplement: S4 Table — (DOCX) [file pone.0158003.s004.docx]

**S4 Table:** Qualitative assessment of included cohort studies according to the quality subscales of the Newcatle-Ottowa scale.

| Cohort | Quality assessment score:  Total (max. 8 points) | Score for subscale:  Selection (max. 4 points) | Score for subscale:  Comparability (max. 1 point) | Score for subscale:  Outcome (max. 3 points) |
| --- | --- | --- | --- | --- |
| *Dirx et al, 1999* | 7 | 3 | 1 | 3 |
| *Dirx et al, 2001* | 7 | 3 | 1 | 3 |
| *Elias et al, 2004* | 6 | 2 | 1 | 3 |
| *Fentiman et al, 2007* | 6 | 3 | 1 | 2 |
| *Keinan-Boker et al, 2009* | 7 | 3 | 1 | 3 |
| *Koupil et al, 2009* | 6 | 3 | 1 | 2 |
| *Robsahm et al, 2009* | 6 | 3 | 1 | 2 |
| *Hughes et al, 2010* | 7 | 3 | 1 | 3 |
| *Heinen et al, 2011* | 7 | 3 | 1 | 3 |
| *Schouten et al, 2011* | 7 | 3 | 1 | 3 |
| *Li et al, 2012* | 6 | 3 | 1 | 2 |

Abbreviations: max., maximum.
